# Clinical isolates of the modern *Mycobacterium tuberculosis* lineage 4 evade host defense in human macrophages through eluding IL-1β-induced autophagy

**DOI:** 10.1038/s41419-018-0640-8

**Published:** 2018-05-24

**Authors:** Alessandra Romagnoli, Elisa Petruccioli, Ivana Palucci, Serena Camassa, Elisabetta Carata, Linda Petrone, Stefania Mariano, Michela Sali, Luciana Dini, Enrico Girardi, Giovanni Delogu, Delia Goletti, Gian Maria Fimia

**Affiliations:** 1 Department of Epidemiology and Preclinical Research, National Institutes for Infectious Diseases Lazzaro Spallanzani IRCCS, Rome, 00149 Italy; 2 Institute of Microbiology, Universita’ Cattolica del Sacro Cuore – Fondazione Policlinico Gemelli, Rome, 00168 Italy; 3 Dipartimento di Scienze e Tecnologie Biologiche ed Ambientali, University of Salento, Lecce, 73100 Italy

## Abstract

*Mycobacterium tuberculosis* (*Mtb*), the causative agent of tuberculosis (TB), has infected over 1.7 billion people worldwide and causes 1.4 million deaths annually. Recently, genome sequence analysis has allowed the reconstruction of Mycobacterium tuberculosis complex (MTBC) evolution, with the identification of seven phylogeographic lineages: four referred to as evolutionarily “ancient”, and three “modern”. The MTBC strains belonging to “modern” lineages appear to show enhanced virulence that may have warranted improved transmission in humans over ancient lineages through molecular mechanisms that remain to be fully characterized. To evaluate the impact of MTBC genetic diversity on the innate immune response, we analyzed intracellular bacterial replication, inflammatory cytokine levels, and autophagy response in human primary macrophages infected with MTBC clinical isolates belonging to the ancient lineages 1 and 5, and the modern lineage 4. We show that, when compared to ancient lineage 1 and 5, MTBC strains belonging to modern lineage 4 show a higher rate of replication, associated to a significant production of proinflammatory cytokines (IL-1β, IL-6, and TNF-α) and induction of a functional autophagy process. Interestingly, we found that the increased autophagic flux observed in macrophages infected with modern MTBC is due to an autocrine activity of the proinflammatory cytokine IL-1β, since autophagosome maturation is blocked by an interleukin-1 receptor antagonist. Unexpectedly, IL-1β-induced autophagy is not disadvantageous for the survival of modern *Mtb* strains, which reside within Rab5-positive phagosomal vesicles and avoid autophagosome engulfment. Altogether, these results suggest that autophagy triggered by inflammatory cytokines is compatible with a high rate of intracellular bacilli replication and may therefore contribute to the increased pathogenicity of the modern MTBC lineages.

## Introduction

*Mycobacterium tuberculosis* (*Mtb*) emerged as a human pathogen 75–150 thousand years ago and spread by clonal expansion among human communities since then, giving rise to seven phylogeographic lineages which show a relatively limited genetic variability, consisting of large-sequence polymorphisms (LSPs) and single-nucleotide polymorphisms (SNPs)^1–3^. The lineages present in the *Mtb* complex (MTBC) are distinguished as ancient and modern. The ancient ones comprise two *Mtb* lineages (lineage 1 and the recently identified lineage 7) causing tuberculosis (TB) in humans, and two *M. africanum* lineages, one of which includes bacilli responsible for TB in animal species^4^. The modern lineages 2, 3, and 4 consist of *Mtb* strains responsible for the largest and deadliest epidemics of the last few centuries and that are driving the current pandemics in Sub-Saharan Africa, Southeast Asia, and Eastern Europe, while the ancient lineage 1 causes TB mainly in the Indo-Oceanic regions and the Philippines^5^. Despite the limited genetic variability within MTBC, the strains belonging to different lineages show significant differences in terms of pathogenicity, transmissibility, and host specificity, as highlighted by the differences between *M. tuberculosis*, *M. africanum*, and *M. bovis* strains. Even human-adapted MTBC strains (*Mtb*) show phenotypic differences in terms of intracellular survival and transcriptional profile in macrophages^6–10^ and virulence in mice^9,10^. The impact of these phenotypic differences between the human-adapted strains on TB disease and transmission remains elusive, although the observed sympatric association between *Mtb* lineages and ethnic human groups supported the continuous adaptive process between *Mtb* and its host^11,12^.

The balance between immune activation and inflammation influences the outcome of *Mtb* infection. Interferon-γ (IFN-γ) and tumor necrosis factor-α (TNF-α), mainly produced by type-1 T helper cells, are key cytokines to contrast *Mtb* infection^13,14^. IFN-γ production is regulated by interleukin (IL) 12, which is secreted by activated phagocytes. Other important proinflammatory cytokines are IL-1β and IL-6, which may lead to the Th17-differentiation, as a subset of T cells essential for the formation of mature pulmonary granulomas^13,15^. Although both inflammatory and adaptive responses are crucial for contrasting mycobacteria infection^16–18^, the extensive immune activation may be self-defeating for the host, paving the way to active TB development and aggravation^13,19,20^.

*Mtb* has developed elaborate survival mechanisms to persist in the host cells, being able to avoid the immune system but, at the same time, chronically stimulating it. The main immune evasion strategies adopted by *Mtb* are based on the inhibition of (a) phagolysosomes formation; (b) antigen processing and presentation to escape from T-cell surveillance; (c) IFN-γ-signaling pathway; and (d) autophagy^15,21–25^.

The role of autophagy during *Mtb* infection has been extensively investigated in the last few years. It is well-known that, besides controlling the turnover of intracellular components, autophagy captures invading pathogens and delivers them to the lysosome for elimination^26^. In this process, target materials are engulfed by double-membrane vesicles, termed autophagosomes, which eventually fuse to lysosomes^27,28^. A specific set of genes, called ATGs, are dedicated to the regulation of autophagy^29^. Among them, members of the Atg8 family, e.g., LC3, are required for autophagosomal membranes expansion and closure, as for the selective recognition of autophagy substrates, including viruses and bacteria, by interacting with adaptor proteins, such as the sequestosome 1/p62-like receptors, which directly bind to cargos^30^.

Both the innate and adaptive responses required a functional autophagic process in order to accomplish their goals^31,32^. Accordingly, autophagy is strictly regulated by a variety of immune sensors, such as Toll-like receptors, Nod-like receptors, IRGM, and nucleic-acid sensors^33^. Moreover, proinflammatory Th1 cytokines, such as IFN-γ, TNF-α, and IL-1β, induce autophagy, while anti-inflammatory Th2 cytokines, such as IL-4 and IL-13 are inhibitory^34,35^. On the other hand, autophagy generally plays an inhibitory role on inflammation, either by eliminating the infectious agent or by physical interaction of ATG proteins with components of the inflammatory pathways, contributing in this way to temporally limit the inflammation response^26^.

Several evidences show that *Mtb* can avoid autophagic degradation and may actually exploit this process to its own advantage by using multiple strategies^36,37^. The type-VII secretion system ESX-1 plays a central role in the modulation of autophagy by *Mtb* H37Rv, being responsible for a rapid activation of autophagy by bacterial DNA through the cytosolic DNA sensor STING^38,39^, as well as for the inhibition of autophagosome–lysosome fusion when infection is established^40,41^. Importantly, autophagy stimulation mediated by cytokines and T cells is able to rescue the block imposed by *Mtb* and favors its killing^36,41^.

Unexpectedly, recent studies show that, apart from ATG5, autophagy-deficient mice do not show increased susceptibility to *Mtb*^42^. Although these data appear to diminish the contribution of basal autophagy in controlling *Mtb*, they are consistent with the reported ability of this bacterium to effectively inhibit autophagy during infection^25^.

Since most of the studies on the host immune response to *Mtb* infection have been performed using the laboratory strain H37Rv, we decided to characterize whether genetic variability of *Mtb* may account for a different ability to modulate the inflammatory response and autophagy flux in primary macrophages.

## Results

### Selection of *MTB*C strains

**Fig. 1:**
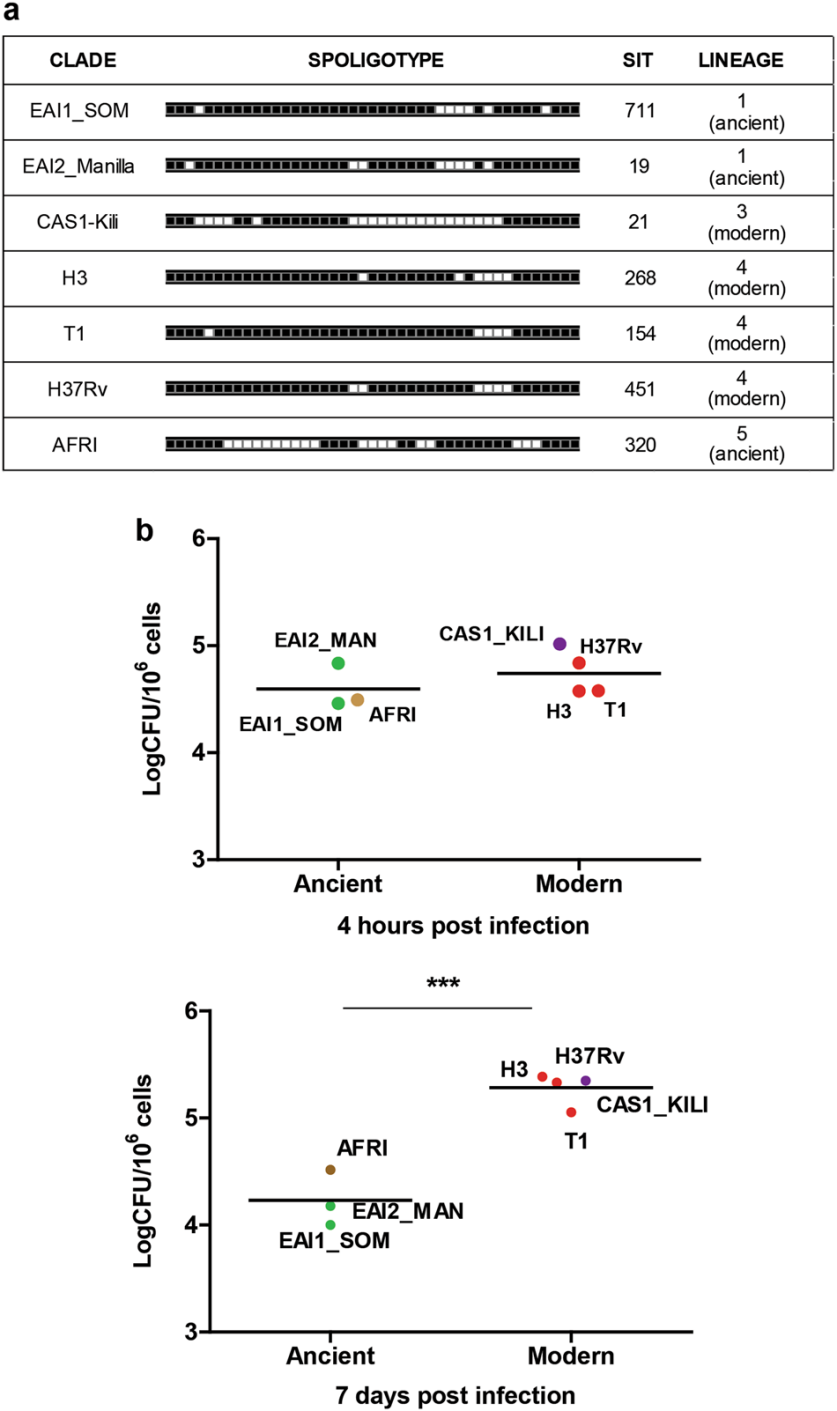
Genetic characterization and replication properties of MTBC strains from ancient and modern lineages in human primary macrophages. **a** Characterization of MTBC lineages by spoligotyping. **b** Macrophages were infected with the indicated MTBC strains. Four hours (upper panel) and 7 days (lower panel) after infection, cells were lysed to measure the number of viable bacteria by plating for determining CFU. Values are expressed as a mean of three independent experiments

To investigate the impact of MTBC genetic diversity on intracellular growth, inflammatory response, and autophagy, six MTBC strains were randomly chosen from a collection of clinical strains based on their genotype determined by spoligotyping (Fig. 1a). We selected two *Mtb* strains of the ancient phylogeographic lineage 1, belonging to clades EAI1_SOM and EAI2_Manilla, and a strain of *M. africanum*, belonging to the ancient lineage 5, which includes strains causing TB in a restricted area of West Africa or in recent immigrants from those regions^5,43^. Two strains belonging to the highly successful phylogeographic lineage 4, of clades H3 and T1, and one strain from lineage 3, clade CAS1-Kili, were selected as representative of the modern *Mtb* strains. We also included the *Mtb* reference strain H37Rv, that, while belonging to the modern lineage 4, has been subjected to many in vitro passages that may have altered its virulence properties^44^. These strains were chosen because they may fairly represent the genetic diversity of MTBC at the global level.

### *Mtb* strains belonging to the modern lineage show a higher rate of intracellular replication

To characterize the virulence properties of the selected MTBC strains, human monocyte-derived macrophages (MDM) were infected at MOI 1:1 and intracellular CFUs were determined at 4 h and 7 days post infection. As shown in Fig. 1b (upper panel), no significant differences were observed in the intracellular CFUs at 4 h post infection, indicating a similar ability of ancient and modern strains to gain access to macrophages. Analysis of the intracellular CFUs at day 7 post infection (Fig. 1b, lower panel) indicated a poor capacity of the two EAI strains to survive and replicate intracellularly and also the *M. africanum* strain showed limited ability to persist in macrophages. Conversely, all *Mtb* strains of the modern clades were capable of replicating intracellularly, with a similar level of intracellular CFUs. Macrophages infected with different *Mtb* strains showed similar viability, as indicated by evaluating LDH release (data not shown), indicating that cytotoxicity may not account for the observed differences of CFUs.

Taken together, these results indicate that the MTBC strains of the modern clades show enhanced ability to replicate intracellularly compared to the strains belonging to the ancient clades.

### *Mtb* strains of modern lineages produce higher levels of inflammatory cytokines

**Fig. 2:**
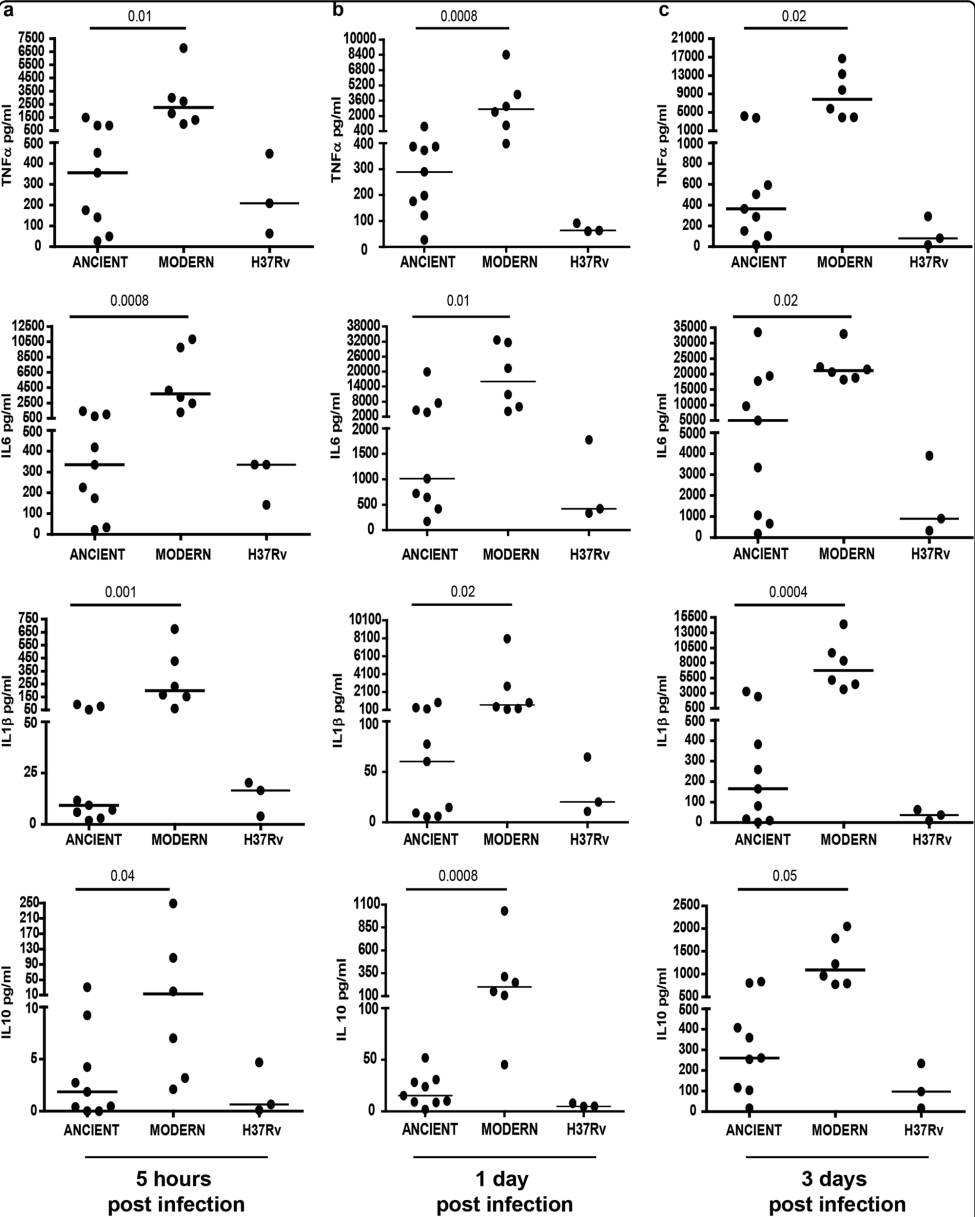
Evaluation of inflammatory cytokine production in human primary macrophages infected with MTBC strains of ancient and modern lineages. Cell culture supernatants were collected from macrophages infected with MTBC (MOI 1:1) at 5 h (**a**), 1 (**b**), and 3 (**c**) days. The production of TNF-α, IL-6, IL-1β, and IL-10 was measured by CBA Flex Set. The results are represented as median of data obtained from modern (H3 and T1) and ancient (EAI1_SOM, EAI2_MANILLA) *Mtb* strains. Data obtained from individual MTBC strain infections are reported in Figure S1

The levels of inflammatory cytokines upon infection of human primary macrophages with modern and ancient MTBC strains were evaluated by cytometry. As shown in Fig. 2 and S1, infection with the modern lineage strains induced a higher production of TNF-α, IL-6, IL-1β, and IL-10 compared to the ancient strains at 5 h (Fig. 2a: *p* = 0.01, *p* = 0.0008, *p* = 0.001, and *p* = 0.04, respectively), 1 day (Fig. 2b: *p* = 0.0008, *p* = 0.01, *p* = 0.02, and *p* = 0.0008, respectively) and 3 days post infection (Fig. 2c: *p* = 0.02, *p* = 0.02, *p* = 0.0004, and *p* = 0.05, respectively).

These data indicate that a proinflammatory signature is induced by macrophage infection with modern *Mtb* strains.

### *Mtb* strains of the modern lineage 4 induce a functional autophagy flux

**Fig. 3:**
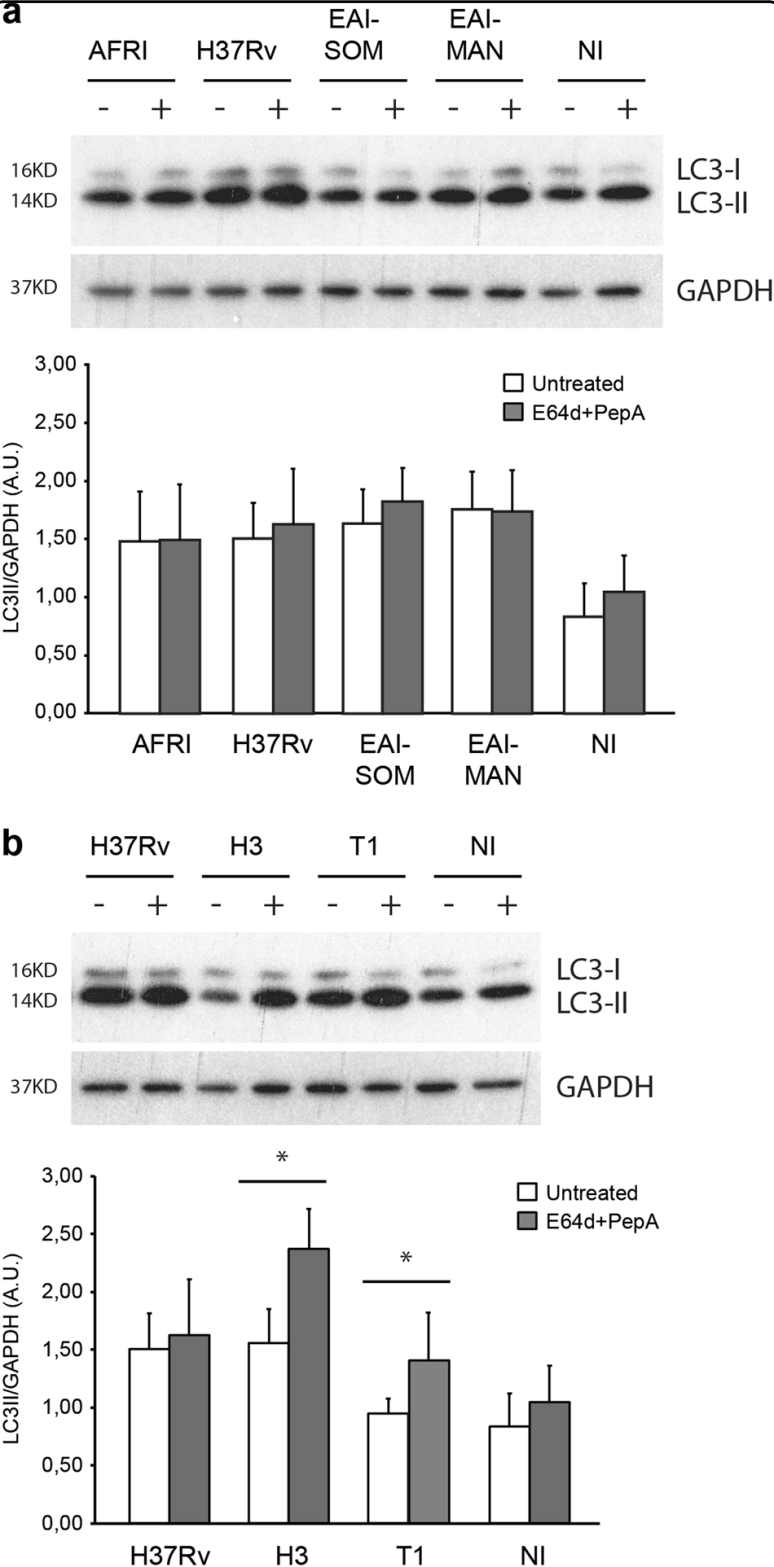
Analysis of autophagy flux in human primary macrophages infected with MTBC strains of ancient and modern lineages. Macrophages were infected with the indicated ancient (**a**) or modern (**b**) MTBC strains and with H37Rv as a reference strain. After 20 h, cells were either incubated with E64d + PepA to inhibit lysosome activity or left untreated. Autophagy levels were analyzed for LC3 expression by immunoblotting. GAPDH levels were analyzed to normalize the amount of protein loaded (upper panels). LC3-II/GAPDH ratios were quantified by densitometric analysis using the ImageQuant software. Each point value represents the mean ± SD from three independent experiments (lower panels). NI: not infected

Autophagy flux was analyzed in human primary macrophages infected with the different *Mtb* strains described in Fig. 1a by comparing the levels of the autophagosome protein LC3-II in the presence or absence of lysosome inhibitors. In macrophages infected for 24 h with the ancient *Mtb* strains, EAI1_SOM, EAI2_Manilla (lineage 1), and AFRI (lineage 5), the inhibition of lysosomal activity does not result in a significant accumulation of LC3-II compared to the untreated cells, indicating that the fusion of autophagosomes with the lysosomes is impaired (Fig. 3a), similar to what was previously reported for the laboratory strain H37Rv^40,41^. Conversely, in macrophages infected with the modern *Mtb* strains H3 and T1, the lysosome inhibitors significantly increased LC3-II levels compared to the untreated cells (Fig. 3b), indicating that newly formed autophagosomes are efficiently delivered to the lysosomes in these cells. Similar results were obtained when autophagic flux was analyzed at 5 h after infection with ancient and modern *Mtb* strains (Figure S2a). Autophagy induction following infection was prevented by using the phosphatidylinositol 3-kinase inhibitor wortmannin, indicating that modern *Mtb* strains trigger a canonical autophagic pathway (Figure S2b).

### The *Mtb* H3 strain of lineage 4 evades the autophagy sequestration

Considering the important contribution of autophagy in *Mtb* clearance^24^, the increased autophagy flux observed in macrophages infected with the modern strains appeared to be in contrast with their high rate of replication. These data prompted us to investigate whether modern *Mtb* strains are spared from the autophagosome-mediated degradation, despite their ability to trigger autophagy.

**Fig. 4:**
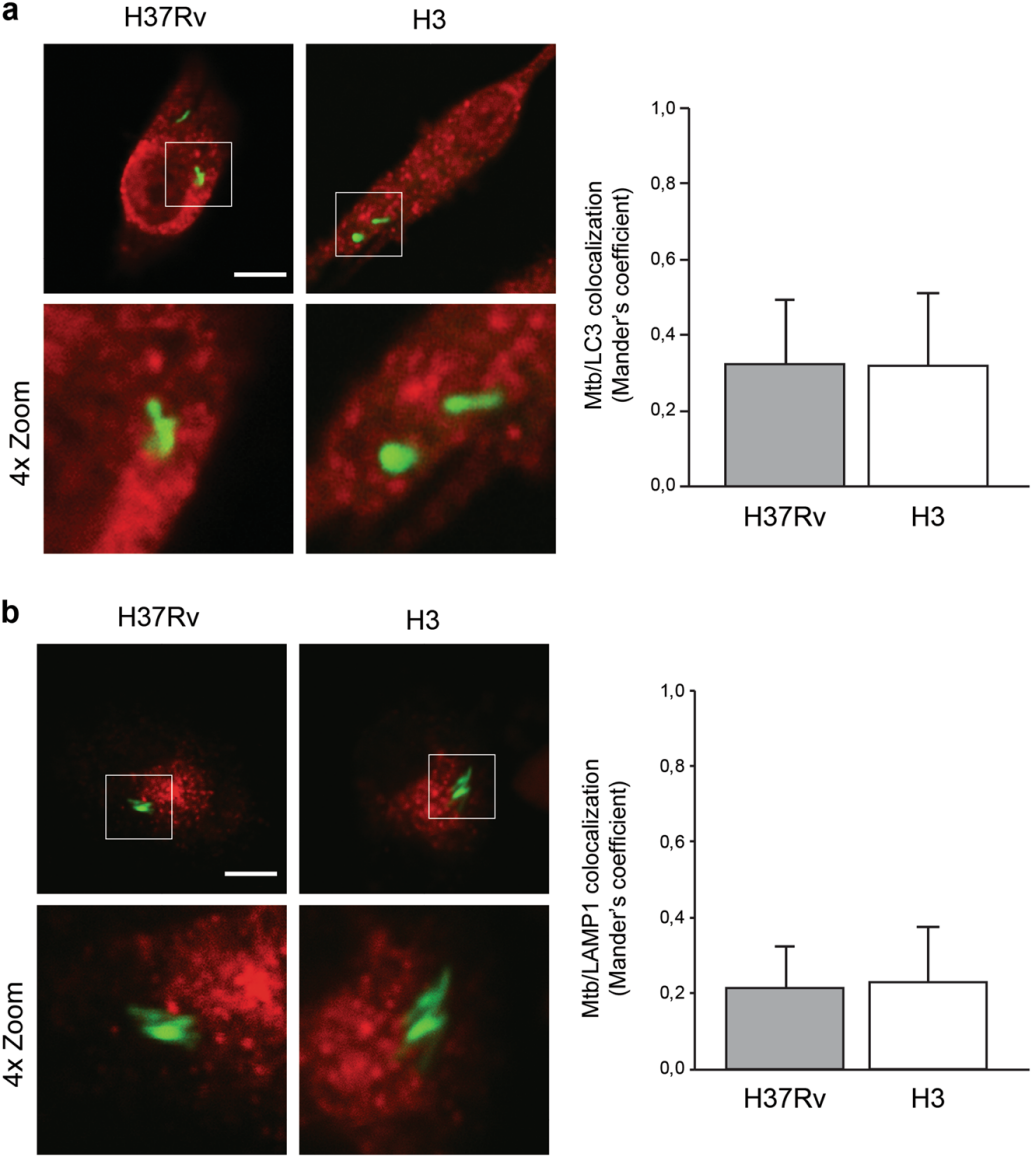
Analysis of *Mtb* H3 localization within autophagosomal/autolysosomal vesicles. Primary macrophages were infected with H3 and H37Rv *Mtb*. Cells were fixed and analyzed for LC3 (**a**) or LAMP1 (**b**) localization by immunofluorescence using specific antibodies, while *Mtb* was detected by auramine staining. The images show that the merging of the two fluorescence signals is shown on the left panels. Green: *Mtb*; red: LC3 in (**a**), LAMP1 in (**b**). Scale bar, 6 μm. Colocalization rate was measured by Mander’s coefficient calculated by ImageJ software. Graphics reporting a quantification of the experiments is shown in the right panels. The results represent the mean ± SD of three independent experiments

To this aim, confocal analysis was carried out to evaluate the colocalization of mycobacteria with the autophagic marker LC3, as well as with ubiquitin and the ubiquitin-binding autophagy adaptors NDP52 and NBR1, which have been reported to mediate the engulfment of *Mtb* in autophagosomes^39,45^. *Mtb* H3 was chosen as a representative strain for the modern lineage and compared to H37Rv. As shown in Fig. 4a and S3, *Mtb* H3 shows low levels of colocalization with LC3, ubiquitin, NDP52 and NBR1, indicating that *Mtb* H3 is not targeted by autophagy.

Moreover, we tested the ability of *Mtb* H3 to prevent the lysosomal degradation, which may occur by the maturation of either phagosomal or autophagosomal compartments. As shown in Fig. 4b, *Mtb* H3 showed a low level of colocalization with the lysosomal marker LAMP1, similar to what was observed in H37Rv-infected cells.

Altogether, our data indicate that, although macrophage infection with modern *Mtb* strains leads to an increased autophagy flux, these bacteria are able to elude the autophagy machinery.

**Fig. 5:**
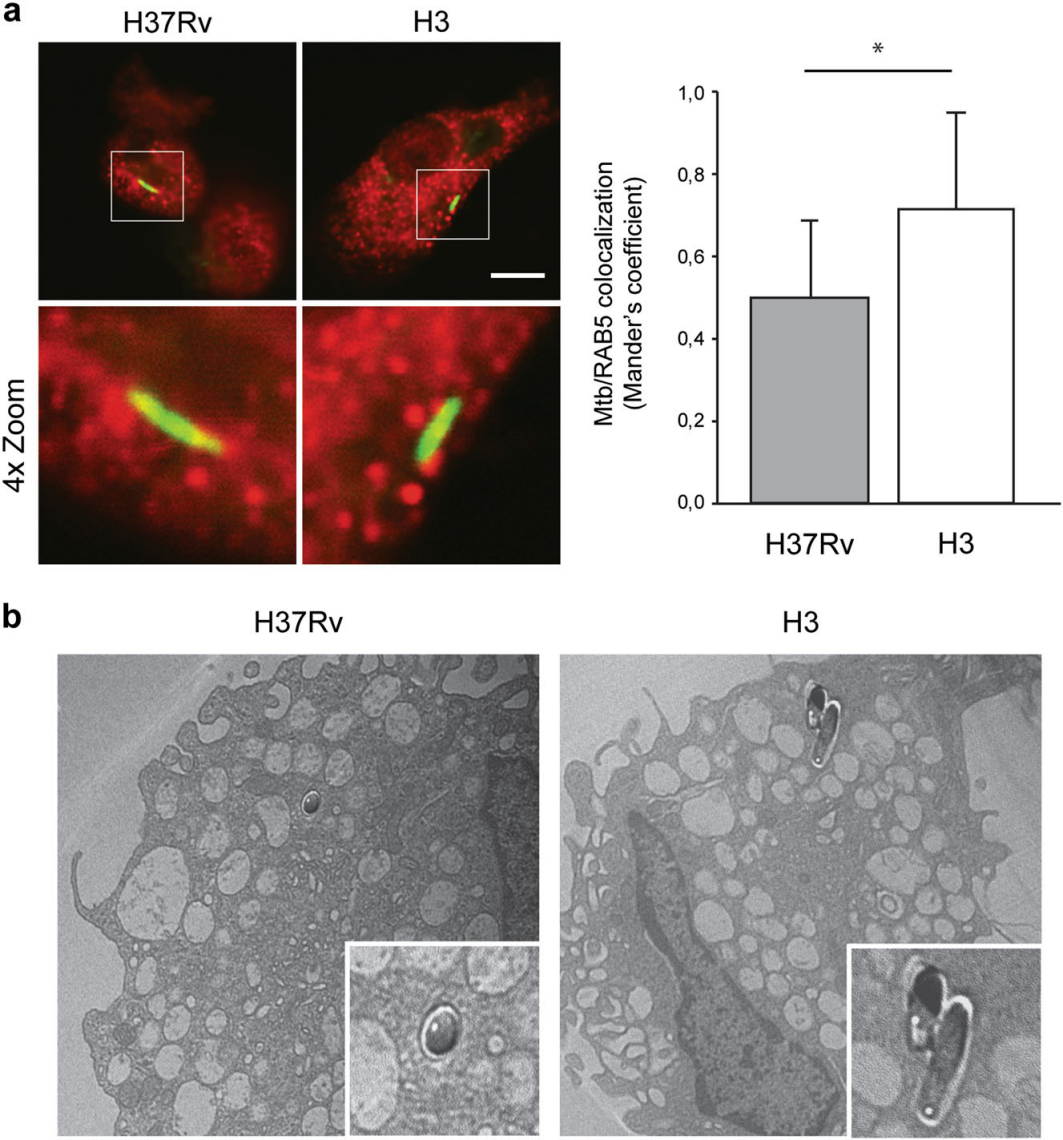
Analysis of localization *Mtb* H3 within the endosomal compartment. **a** Primary macrophages were infected with H3 and H37Rv *Mtb*. Cells were fixed and analyzed for Rab5 localization by immunofluorescence using a specific antibody, while *Mtb* was detected by auramine staining (left panel). The images show that the merging of the two fluorescence signals is shown on the left panels. Green: *Mtb*; red: RAB5. Scale bar, 6 μm. Colocalization rate was measured by Mander’s coefficient calculated by Image J software. Graphics reporting a quantification of the experiments is shown in the right panel. The results represent the mean ± SD of three independent experiments. **b** Ultrastructural analysis of *Mtb* H37Rv and H3-infected macrophages 24 h post infection. Mycobacteria are indicated by asterisks (×1.500). A magnification of *Mtb*-containing vesicles is shown in the panels below (×4.000)

Recently, *Mtb* strains belonging to the modern lineages were reported to have a preferential cytosolic residence in the infected macrophages, which was proposed as an alternative adaptation mechanism to avoid the autophagy degradation^46^. Thus, we investigated if the autophagy-resistant properties of the modern *Mtb* H3 were due to an increased cytosolic localization. To this aim, confocal analyses were performed to evaluate the localization of H37Rv and H3 mycobacteria within Rab5-positive or CD63-positive endosomal/phagosomal compartments (Fig. 5a and S4). Our data show that *Mtb* H3 has a predominant phagosomal localization, similar to what was observed in H37Rv-infected cells. Phagosomal localization of *Mtb* H3 was also confirmed by performing electron microscopy analysis (Fig. 5b).

Taken together, these results suggest that, similar to H37Rv, *Mtb* H3 is able to prevent its autophagosomal engulfment by hiding within the phagosomes.

### *Mtb* H3 strain of lineage 4 induces a functional autophagy flux through IL-1β-dependent signaling

Our data show that modern *Mtb* strains of lineage 4 induce high levels of IL-1β production and autophagy. Since IL-1β has been demonstrated to be a powerful inducer of autophagy in human macrophages^47^, we investigated the functional relationship between IL-1β and autophagy in *Mtb* H3-infected macrophages.

**Fig. 6:**
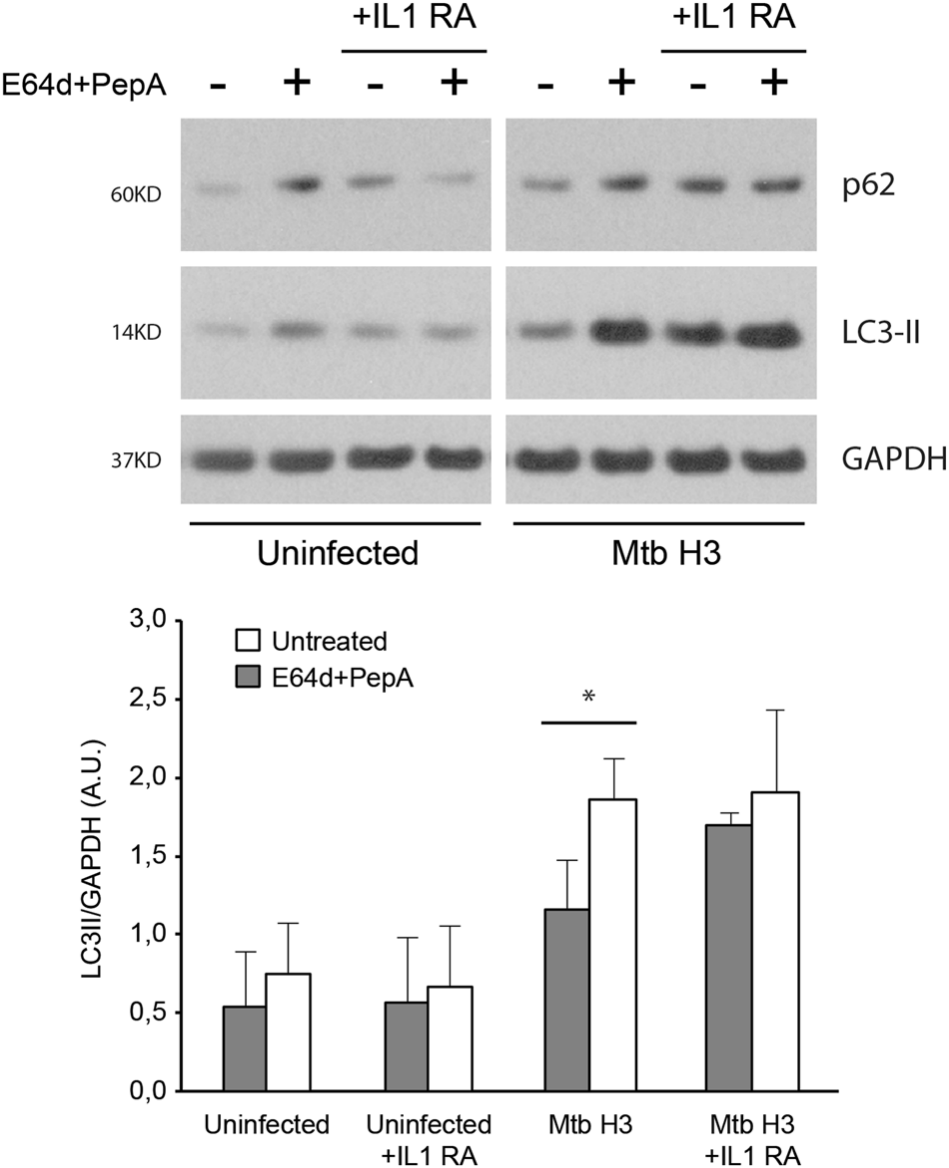
Role of IL-1β on the induction of autophagy triggered by *Mtb* H3. Macrophages were infected with the *Mtb* H3 and H37Rv strains. After 2 h, uninfected and infected cells were treated with an IL-1β receptor antagonist or left untreated. After 20 h, cells were incubated with E64d + PepA to inhibit lysosome activity or left untreated. Autophagy levels were analyzed by evaluating LC3 and p62 expression by immunoblotting. GAPDH levels were analyzed to verify protein amount loading (upper panels). LC3-II/GAPDH ratios were quantified by densitometric analysis using the ImageQuant software. Each point value represents the mean ± SD from three independent experiments (lower panel)

To this aim, IL-1β activity was blocked using an IL-1 receptor antagonist (IL-1-RA) and autophagy flux evaluated by western blot analysis. Notably, IL-1-RA treatment inhibited the autophagic flux in macrophages infected with *Mtb* H3, as shown by the reduced increase of the autophagy markers LC3-II and p62 observed after the inhibition of the lysosomal activity (Fig. 6). According to the capability of *Mtb* H3 to escape the autophagic response, the inhibition of either IL-1β signaling by IL-1-RA or the IL-1β-induced autophagic flux by a lysosome inhibitor did not have an impact on bacterial growth in infected macrophages (Figure S5).

These data suggest that the different ability of ancient and modern *Mtb* to modulate the autophagy flux in macrophages is associated with their inflammatory properties.

## Discussion

Recent studies have shown the presence of lineage-specific effects on the outcome of *Mtb* infection and disease^11^. However, how the differences of *Mtb* genomes impact on the host response remains poorly characterized. Here, we compared ancient and modern MTBC strains with regard to their ability to replicate in human macrophages and to modulate innate immune responses in terms of proinflammatory cytokines and autophagy. We found that strains of the modern lineages 3 and 4 have an improved ability to grow in human primary infected macrophages compared to strains of the ancient lineages 1 and 5. These results are in line with previous findings showing an enhanced intracellular survival and replication rate of the strains belonging to the modern lineages when compared to the reference strains (H37Rv and CDC1551) and the MTBC strains of the ancient lineages (EAI and *M. africanum*), which showed an even less efficient intracellular survival^6,10^. However, it should be noted that differences in the intracellular replication rate have also been observed between strains belonging to the same lineage, indicating the presence of both intra-lineage or intra-genotype variability^48,49^.

Unexpectedly, a more efficient intracellular replication is paralleled by an increased production of inflammatory cytokines and higher levels of autophagy. In detail, we found that TNF-α, IL-1β, IL-6, and IL-10 levels are higher in H3 and T1 strains of lineage 4 than in the two EAI strains from lineage 1 and *M. africanum*. To note, the anti-inflammatory cytokine IL-10 is also increased after H3 and T1 infection. This observation is in agreement with the important role proposed for this cytokine in counterbalancing an excessive innate and adaptive immune response against Mtb, and could represent an important target to alter the survival rate of the modern Mtb strains in macrophages^16,50^.

It has been previously reported that mycobacteria belonging to the modern lineages induce a lower immune response compared to the ancient ones^8^. In fact, Portevin and colleagues observed a reduction of proinflammatory cytokine levels in both macrophages, monocyte-derived dendritic cells, and murine bone marrow-derived macrophages. Possible differences that may account for this discrepancy could be represented by the procedure we used for the ex vivo differentiation of macrophages, which does not include GM-CSF^8^, or the difference in the clinical isolates tested^51^. In fact, it has been observed that distinct clinical isolates from the same lineages may differently modulate the immune response^51^.

Activation of proinflammatory responses by *Mtb* infection depends on the binding of bacterial components to extracellular PRRs^52^ and to the intracellular activation of the NLRP3 inflammasome^53^. It has been shown that differences in surface lipids, sugars, and protein compositions among *Mtb* strains may impact the innate immune responses^54–56^. Moreover, intracellular pathogen recognition pathways, including IL-1β production via the NLRP-3 inflammasome and type-I IFNs via the cGAS/STING pathway, can be differently stimulated according to the level of secreted EsxA^57^. It follows that differential expression or polymorphisms affecting ESX-1 may have an impact on IL-1β secretion, probably by influencing the translocation of proteins and other *Mtb* metabolites to the cytosol of infected macrophages^58^. However, since MTBC strains belonging to modern and ancient lineages were shown to differentially induce type-I IFN, despite the fact that these strains were equally capable of accessing the cytosol^59^, it is likely that multiple factors may contribute to the inflammatory response to different MTBC strains, as, for example, the extent of the mitochondrial damage.

Activation of IL-1β has been previously shown to promote *Mtb* killing through the activation of the TNF-α signaling, which culminates with caspase-3-induced apoptosis followed by the uptake and the lysosomal degradation of *Mtb*-containing apoptotic bodies by uninfected neighbors, a process defined as efferocytosis^60,61^. In addition, IL-1β is also known to induce autophagy that in turn has been shown to restrict *Mtb* replication in macrophages^47^. In line with these observations, the H3 and T1 strains, which trigger a higher level of IL-1β compared with ancient and reference strains, induce a functional autophagic process, as shown by analyzing the degradation rate of the autophagosomal marker LC3II. Conversely, cells infected with ancient MTBC strains and H37Rv, secrete lower levels of IL-1β and show an inhibited autophagic flux. Notably, we found that the increased production of IL-1β was indeed responsible for the induction of autophagy, since an inhibitor of IL-1β prevented autophagy flux in H3-infected macrophages, reestablishing the block of autophagosome maturation observed with H37Rv and the ancient strains. However, despite the higher autophagy induction and consistent with its increased ability to survive in macrophages, *Mtb* H3 strain is able to hide within endosomal compartments and to elude autophagosome engulfment and consequent lysosomal degradation. Understanding the molecular mechanisms that allow modern *Mtb* strains to escape IL-1β-induced autophagy degradation will be relevant to design novel therapeutic strategies aimed at preventing their intracellular survival. The fact that several drugs specifically targeting autophagy, inflammation, and immunometabolism have been shown to exert anti-TB activity highlights the potential consequences of these studies in terms of development of new host-directed therapies against TB^62–64^.

Modulation of IL-1β and autophagy levels may have a relation with the increased virulence observed in strains of modern lineages. In fact, IL-1β is required for protection against TB but a chronic high level of IL-1β is associated with pathology, likely causing a non-protective response^65^. At the same time, IL-1β-induced autophagy could turn out to be beneficial for chronic infection by sustaining macrophages metabolism^66^ and protecting against excessive inflammation during *Mtb* infection^67^. In this line, it has been recently proposed that *Mtb* may selectively regulate autophagy to extend survival in infected macrophages^68^. This hypothesis is also supported by our observation that infection with modern *Mtb* strains does not result in decreased macrophage viability, which would have been expected in cells infected with highly replicating *Mtb* strains. Hence, the ability of modern *Mtb* strains to efficiently replicate intracellularly in macrophages, while inducing autophagy, may represent an advantage acquired during evolution to enhance bacilli persistence in inflammed host tissues. Potential limitations of the present study should be considered. We analyzed a relatively small number of clinical isolates. However, although larger studies are needed to confirm these observations, the data generated here on the modulation of the autophagy process are confirmed by different approaches such as immunoblotting, confocal, and electron microscopy.

In view of these findings, it will be important to test how the different ability of modern *Mtb* strains to alter autophagy and inflammation impacts on the regulation of specific T-cell response. This is expected to uncover new aspects on the complex mechanism that allows *Mtb* to escape the immune response and be protected from vaccinal approaches. The results of this study may have translational implications for the design of new TB vaccines or host-directed therapies, which should take into account both the autophagic and immunogenic characteristics of the lineage of the *Mtb* candidate.

## Materials and methods

### MTBC strains

The MTBC strains used in this study were selected from a collection of clinical strains isolated at the *Università Cattolica* del *Sacro Cuore* in Rome and *Mtb* H37Rv was used as a reference strain. The MTBC strains were grown in Middlebrook 7H9 (Difco BD, NY) medium supplemented with 0.2% glycerol, 10% ADC (bovine albumin fraction V, dextrose, and catalase; Microbiol [Cagliari, Italy]), and 0.05% Tween 80 (Sigma-Aldrich, St. Louis, MO) at 37 °C. Mycobacterial cultures were harvested at late log phase, glycerol was added at 20% final concentration, and 1-ml aliquots were stored at –80 °C. Genomic DNA was extracted from liquid cultures by using the CTAB method, as previously described^69^.

#### Phylogenetic analysis of MTBC strains

The genomic DNA of each MTBC clinical strain was genotyped by spoligotyping^70^. For each experimental session, genomes of *Mtb* H37Rv and *M. bovis* BCG were used as positive controls and water as negative control.

### Macrophage culture

Peripheral blood mononuclear cells (PBMCs) were obtained from healthy donors. PBMCs were isolated by density-gradient centrifugation. Monocytes were purified from PBMCs by positive sorting, using anti-CD14-conjugated magnetic microbeads (Miltenyi Biotec, Auburn, CA). After the selection, the level of purification was verified by flow cytometry (FACS Canto II flow cytometer; Becton Dickinson, Milan, Italy): 99% of CD14 + monocytes and 1% of CD3 + T cells. Macrophage-derived monocytes (MDM) were obtained by cultivating adherent monocytes for 5–6 days in X-Vivo 15 medium (Lonza, Walkersville, MD) and 2% human serum (Euroclone, Paignton, UK) at 37 °C in a 5% humidified atmosphere until macrophage differentiation.

Depending on the experimental requirements, different amount of cells were used. In detail, for the western blot experiments, the cells were seeded in six-well flat-bottom tissue culture plates (3 × 10^6^ cells/well, 2 ml/well; Corning, New York, NJ). For the confocal experiments, cells were seeded in glass slide chambers (Nunc, Lab-Tek, Waltham, Massachusetts, USA) (5 × 10^5^ cells/well, 2 ml/well). For the colony-forming unit (CFU) experiment, cells were seeded in 24-well flat-bottom tissue culture plates (5 × 10^5^ cells/well, 1 ml/well; Corning, New York, NJ). For electron microscopy, cells were seeded in 60-mm tissue culture plates (5.5 × 10^6^ cells; Corning, New York, NJ).

Autophagy was evaluated both 5 and 24 h after infection. To assess the autophagy flux, the lysosomal inhibitors E64d/pepstatin A (5 µg/ml; Sigma-Aldrich, St. Louis, MO), were added 4 h before lysis. Wortmannin (20 nM Sigma-Aldrich, St. Louis, MO) was added 5 h before lysis. To inhibit IL-1b activity, soluble antagonist for IL-1 receptor (IL-1-RA) at 1 µg/ml (R&D, Minneapolis, MN) was added to the macrophage cultures before infection. IL-1-RA was added again after the infection, concomitantly to the medium change; the uninfected cells followed the same treatment^71^.

To evaluate the viability of the MTBC-infected macrophages, the LDH Cytotoxicity Assay Kit (Thermo Fisher, Waltham, MA, USA) was used. The LDH Cytotoxicity Assay Kit is a reliable colorimetric assay to quantitatively measure lactate dehydrogenase (LDH) released into the media from damaged cells as a biomarker for cellular cytotoxicity and cytolysis.

### MTBC infection of macrophages and colony-forming unit assay

Macrophages were infected with different MTBC strains (HR7Rv, EAI1_SOM, EAI2_MANILLA, AFRI, H3, and T1) at a multiplicity of infection (MOI) of 1:1 for western blot analysis and CFU and at a MOI of 2:1 for confocal experiments and electron microscopy. Two hours after the in vitro infection, macrophages were washed once with phosphate-buffered saline (PBS) and then fresh medium was added.

To determine the intracellular bacterial load, CFUs of infected macrophages were measured in triplicate and determined at 4 h and 7 day post infection^55^. Briefly, infected cell cultures were lysed in PBS 0.1%, Triton X (Sigma-Aldrich, St. Louis, MO), and the serial dilution was prepared in PBS 0.05% Tween 80 (Sigma-Aldrich, St. Louis, MO). Fifty-microliter aliquots of each dilution were plated on 7H11/OADC (Difco BD, NY) agar plates. The plates were incubated for 3 weeks.

### Western blot assays

Cells were collected 5 and 24 h post infection and lysed in CelLytic buffer (Sigma-Aldrich, St. Louis, MO) plus the following protease and phosphatase inhibitors: protease inhibitor cocktail, 1 mM sodium fluoride, 1 mM sodium orthovanadate, and 1 mM sodium molybdate; 1 mM phenylmethylsulfonyl fluoride (Sigma-Aldrich, St. Louis, MO). Proteins were resolved on 12% NuPAGE Bis-Tris gel (Life Technologies, Carlsbad, CA) and electroblotted onto PVDF membranes (Millipore, Billerica, MA). Blots were incubated with primary antibodies in 5% nonfat dry milk in PBS plus 0.1% Tween-20 (Sigma-Aldrich, St. Louis, MO) overnight at 4 °C. Proteins were detected using horseradish peroxidase-conjugated secondary antibody (Jackson Laboratory, Bar Harbor, ME) and visualized with ECL plus (GE Healthcare, Little Chalfont, UK).

The primary antibodies used in this study were rabbit anti-LC3 (Cell Signaling, Danvers, MA) (1:2000), mouse anti-p62 (Santa Cruz Biotech, Santa Cruz, CA) (1:1000), and goat anti-GAPDH (Santa Cruz Biotech, Santa Cruz, CA) (1:50,000).

### Confocal microscopy

Twenty-four hours after infection, cells were fixed with 4% paraformaldehyde (Sigma-Aldrich, St. Louis, MO) in PBS followed by permeabilization with 0.2% Triton X-100 (Sigma-Aldrich, St. Louis, MO) in PBS. Mycobacteria were stained with auramine for 20 min and destained for 10 min (TB Fluorescent Stain Kit BD, Sparks, MD). Cells were then labeled with the primary antibody anti-LC3 (L7543, Sigma-Aldrich, St. Louis, MO), anti-LAMP1 (Ab-24170, Abcam, Cambridge, UK) and anti-Rab5a (S-19, Santa Cruz Biotech, Santa Cruz, CA), anti-NDP52 (Ab-184688, Abcam, Cambridge, UK), anti-CD63 (MX-49.129.5, Santa Cruz Biotech, CA), anti-NBR1 (NBP1-71703, Novus Biological, Littleton, Colorado, CA), and anti-ubiquitin (FK2) (ST1200, Millipore, Billerica, MA) for 1 h at room temperature and visualized by means of Cy3-conjugated secondary antibodies (Jackson Immunoresearch). Coverslips were mounted in Prolong Gold antifade (Life Technologies, Carlsbad, CA) and examined under a confocal microscope (Leica TCS SP2, Wetzlar, Germany). Digital images were acquired with the Leica software and the image adjustments and merging were performed by using the appropriated tools of ImageJ software. Quantification of colocalization, expressed in terms of Mander’s coefficient, was calculated using the JacoP plugin of ImageJ software. A minimum of 50 cells per sample experimental condition were counted for triplicate samples per condition in each experiment.

### Cytokine detection

Supernatants from MTBC-infected macrophage cultures were collected at different time points after in vitro infection and cytokines were evaluated by a cytometric bead array (BD Biosciences, San Jose, CA).

### Electron microscopy

Twenty-four hours after infection, cells were fixed on ice with 2.5% glutaraldehyde in 0.1 M cacodylate buffer, pH 7.4, for 120 min and postfixed with 1% osmium tetraoxide for 120 min at 4 °C. After an extensive washing with cacodylate buffer at 0.1 M, pH 7.4, cells were dehydrated in graded concentrations of ethanol (30, 50, 70, 90, and 100%) and finally embedded in Spurr resin (TAAB). Ultrathin sections (60 nm) were subsequently cut with a PT-PC PowerTome ultramicrotome (RMC Boeckeler). Sections, collected on copper grids, were stained with saturated aqueous uranyl acetate and counterstained with 4% lead citrate, and then observed with HT-7700 TEM (Hitachi, Tokyo, Japan).

### Statistical analysis

Data were analyzed by the GraphPad Prism software, version 4.00 for Windows (GraphPad Software, San Diego, CA). For all the experiments shown, with the exception of the cytokine results, the data were evaluated by analyzing the mean and standard deviation. The statistical significance of the differences between two groups was determined using the Student’s t-test. For the cytokine results, data are analyzed evaluating the median and *P*-values were calculated using the Mann–Whitney test. Differences were considered significant if *P*-values were ≤0.05.

## Electronic supplementary material

Supplementary Figure Legends

Supplementary Figure S1

Supplementary Figure S2

Supplementary Figure S3

Supplementary Figure S4

Supplementary Figure S5
