# Supplementary material for: Clinical isolates of the modern Mycobacterium tuberculosis lineage 4 evade host defense in human macrophages through eluding IL-1β-induced autophagy
Source: Cell Death Dis. 2018 May 24;9(6):624. doi: 10.1038/s41419-018-0640-8 (PMC5967325; doi:10.1038/s41419-018-0640-8)
Supplement: Supplementary file 1 — Supplementary Figure Legends [file 41419_2018_640_MOESM1_ESM.docx]

**SUPPLEMENTARY FIGURE LEGENDS**

**Supplementary Fig. S1.** Evaluation of inflammatory cytokine production in human primary macrophages infected with H3, T1, EAI1_SOM, EAI2_MANILLA MTBC strains.

Cell culture supernatants were collected from macrophages infected with MTBC (MOI 1:1) at 5 h (panel **a**), 1 (panel **b**) and 3 (panel **c**) days. The production of TNF-α, IL6, IL1β and IL-10 was measured by CBA Flex Set. Data are from three independent experiments and bars represent the median value. Red dots refer to modern lineages Black dots: refer to ancient lineages.

**Supplementary Fig. S2**. Analysis of autophagy flux in human primary macrophages infected with different MTBC strains.

(**a**) Macrophages were infected with the modern MTBC strain H3, the ancient MTBC strain EAI2_Manilla strains and with H37Rv as a reference strain. After 6 h, cells were either incubated with E64d+PepA to inhibit lysosome activity or left untreated. Autophagy levels were analysed for LC3 expression by immunoblotting. GAPDH levels were analysed to normalize the amount of protein loaded (upper panels). LC3-II/GAPDH ratios were quantified by densitometric analysis using the ImageQuant software. Each point value represents the means ± SD from three independent experiments (lower panels). NI: not infected.

(**b**) Macrophages were infected with the *Mtb* H3 strain. After 18 h, cells were treated with the PI3K inhibitor wortmannin or left untreated. After 20 h, cells were incubated with E64d+PepA to inhibit lysosome activity or left untreated. Autophagy levels were analysed by evaluating LC3II expression by immunoblotting. GAPDH levels were analysed to verify protein amount loading (upper panels). LC3-II/GAPDH ratios were quantified by densitometric analysis using the ImageQuant software. Autophagic flux was determined as the ratio between normalized LC3II values of E64d+PepA-treated and untreated samples.

**Supplementary Fig. S3**. Analysis of *Mtb* H3 localization within autophagosomal/autolysosomal vesicles.

Primary macrophages were infected with H3 and H37Rv *Mtb*. Cells were fixed and analysed for ubiquitin (**a**), NDP52 (**b**), or NBR1 (**c**) localization by immunofluorescence using specific antibodies, while *Mtb* was detected by auramine staining. The images show the merge of the two fluorescence signals are shown on the left panels. Green: *Mtb*; Red: ubiquitin in (**a**), NDP52 in (**b**), NBR1 (**c**). Scale bar, 6μm. Colocalization rate was measured by Mander’s coefficient calculated by Image J software. Graphics reporting a quantification of the experiments are shown in the right panels. The results represent the mean ± SD of three independent experiments.

**Supplementary Fig. S4**. Analysis of localization *Mtb* H3 within CD63-positive endosomal compartment.

(**a**) Primary macrophages were infected with H3 and H37Rv *Mtb*. Cells were fixed and analysed for CD63 localization by immunofluorescence using a specific antibody, while *Mtb* was detected by auramine staining (left panel). The images shows the merge of the two fluorescence signals are shown on the left panels. Green: *Mtb*; Red: RAB5. Scale bar, 6μm. Colocalization rate was measured by Mander’s coefficient calculated by Image J software. Graphics reporting a quantification of the experiments is shown in the right panel. The results represent the mean ± SD of three independent experiments.

**Supplementary Fig. S5**. Impact of IL1β on the intracellular replication of *Mtb* H3.

(**a**) Macrophages were infected with the *Mtb* H3 strain. During and after the infection cells were treated with an IL1β receptor antagonist (IL1-RA) at 1 µg/ml or left untreated. Two days after infection, cells were lysed to measure the number of viable bacteria by plating for determining CFU. Values are expressed as a mean of three independent experiments. (**b**) Macrophages were infected with the *Mtb* H3 strain. Twenty-four hours upon infection, cells were treated with the lysosomal inhibitor Bafilomycin A1 at 5nM for 24 hours, or left untreated. Two days after infection, cells were lysed to measure the number of viable bacteria by plating for determining CFU. Values are expressed as a mean of three independent experiments.
