# Supplementary figures and images for: Clinical isolates of the modern Mycobacterium tuberculosis lineage 4 evade host defense in human macrophages through eluding IL-1β-induced autophagy
Source: Cell Death Dis. 2018 May 24;9(6):624. doi: 10.1038/s41419-018-0640-8 (PMC5967325; doi:10.1038/s41419-018-0640-8)

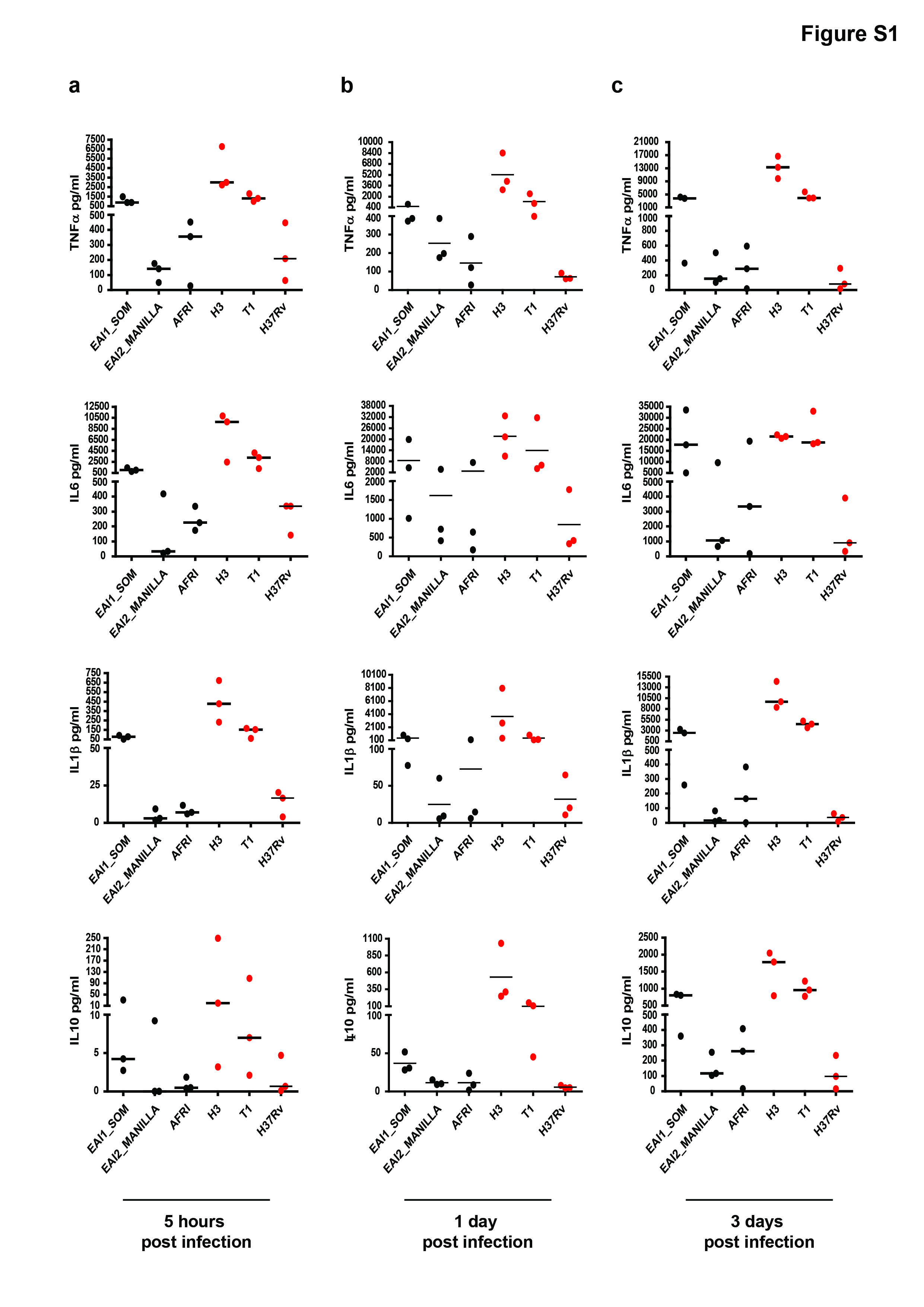

Supplement: Supplementary file 2 — Supplementary Figure S1 [file 41419_2018_640_MOESM2_ESM.tif]

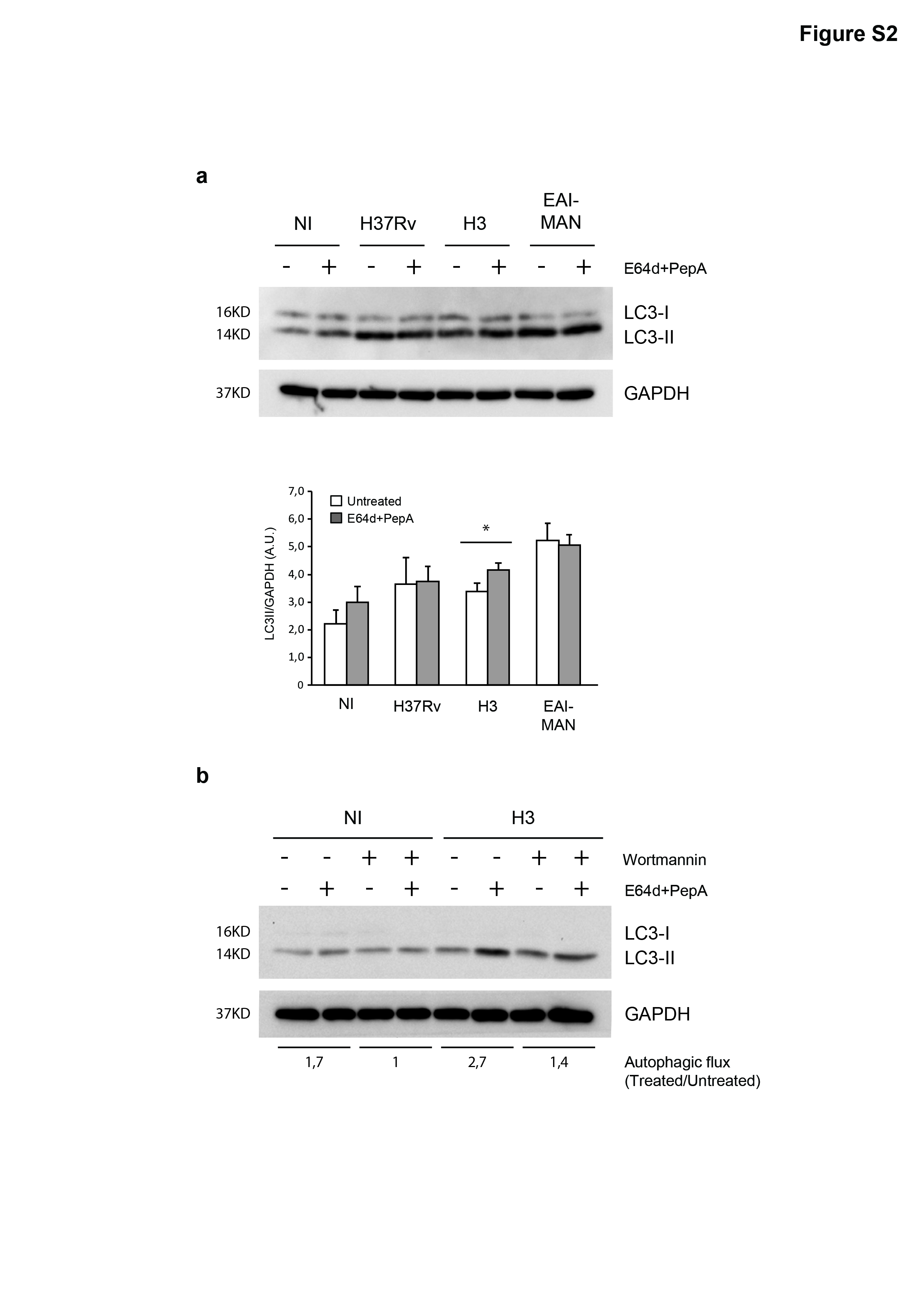

Supplement: Supplementary file 3 — Supplementary Figure S2 [file 41419_2018_640_MOESM3_ESM.tif]

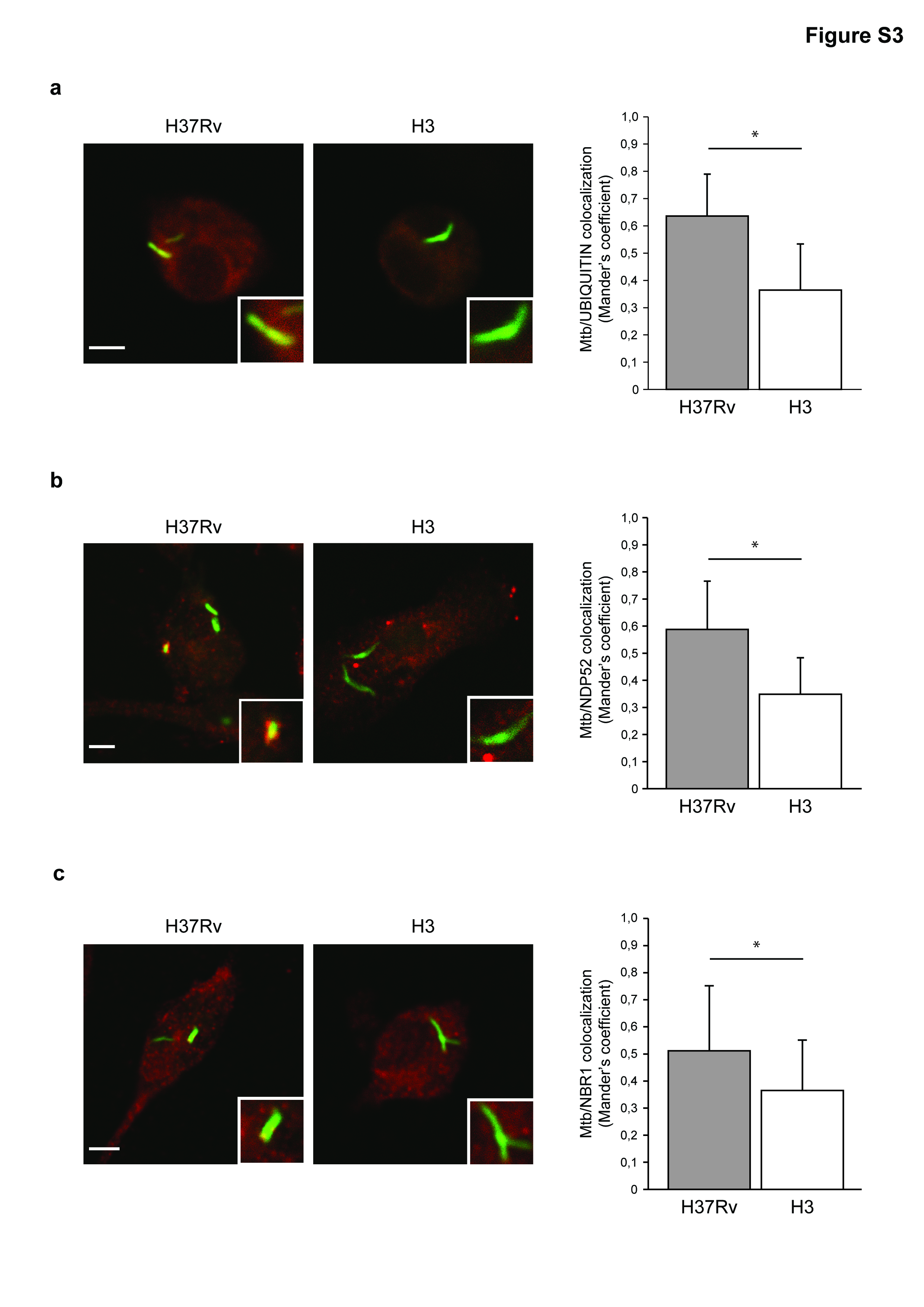

Supplement: Supplementary file 4 — Supplementary Figure S3 [file 41419_2018_640_MOESM4_ESM.tif]

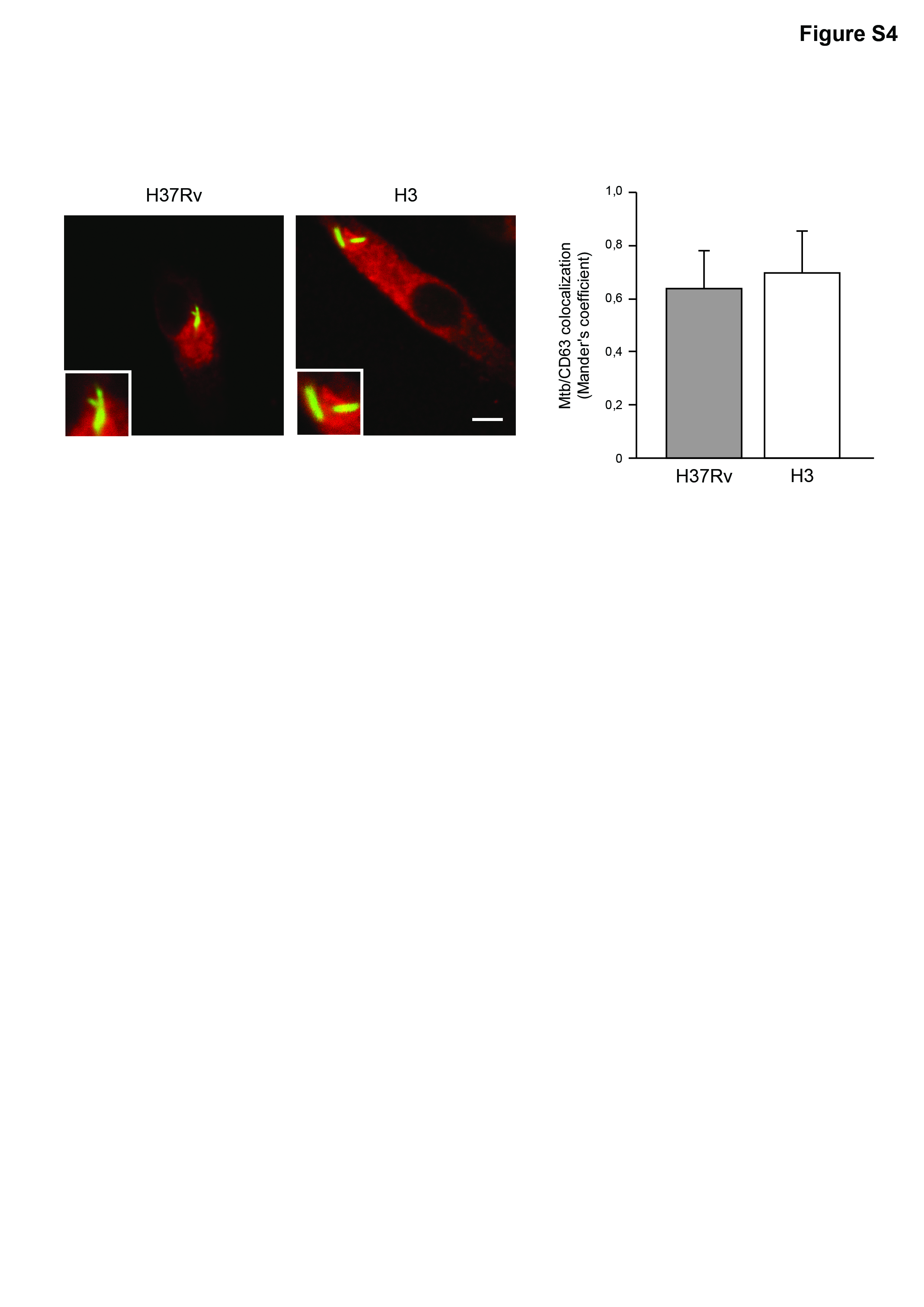

Supplement: Supplementary file 5 — Supplementary Figure S4 [file 41419_2018_640_MOESM5_ESM.tif]

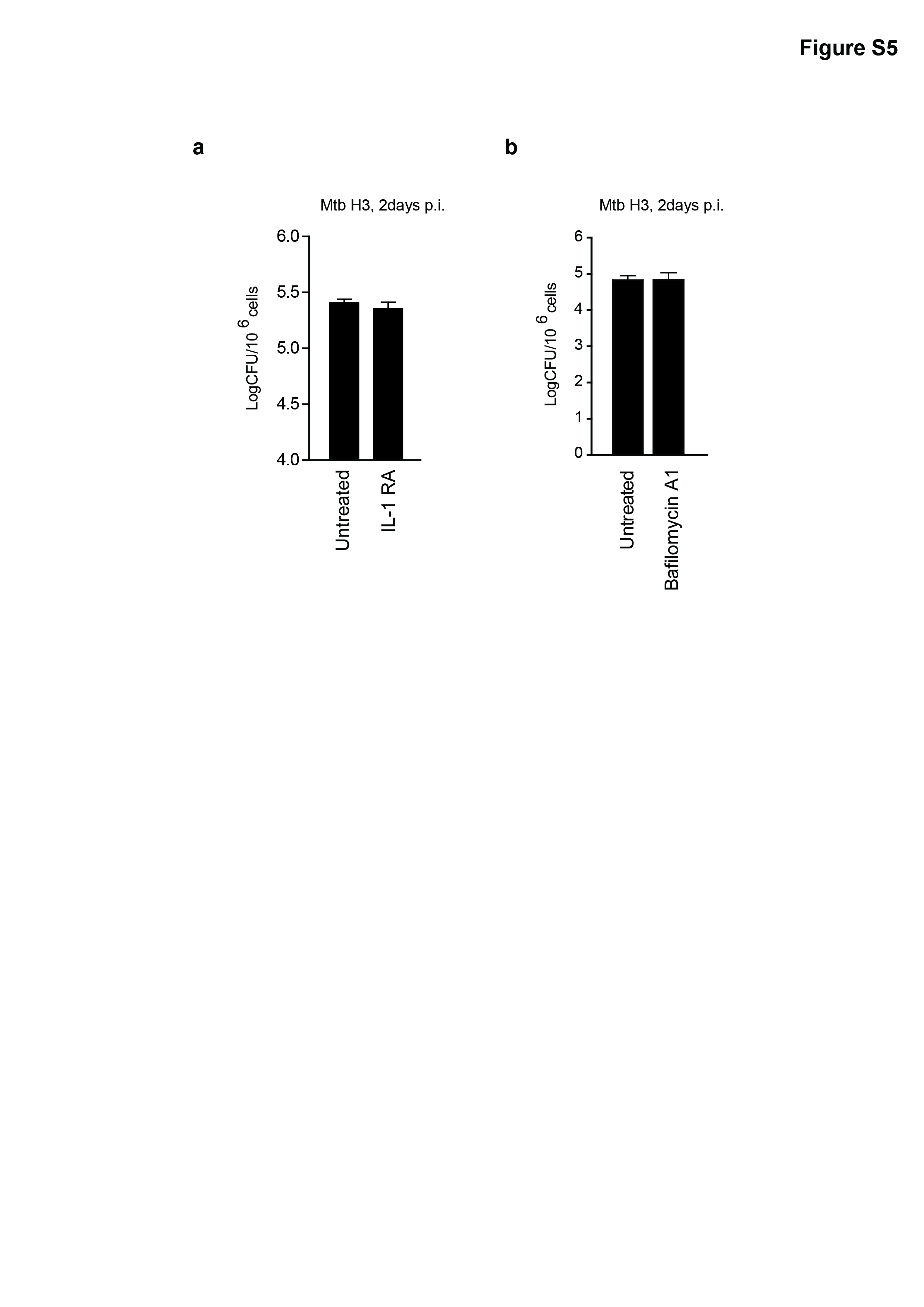

Supplement: Supplementary file 6 — Supplementary Figure S5 [file 41419_2018_640_MOESM6_ESM.tif]
